# Ultrathin Tungsten Oxide Nanowires/Reduced Graphene Oxide Composites for Toluene Sensing

**DOI:** 10.3390/s17102245

**Published:** 2017-09-29

**Authors:** Muhammad Hassan, Zhi-Hua Wang, Wei-Ran Huang, Min-Qiang Li, Jian-Wei Liu, Jia-Fu Chen

**Affiliations:** 1 Hefei National Laboratory for Physical Sciences at the Microscale, Collaborative Innovation Center of Suzhou Nano Science and Technology, Department of Chemistry, University of Science and Technology of China, Hefei 230026, China; mhassan@mail.ustc.edu.cn (M.H.); wzhua@mail.ustc.edu.cn (Z.-H.W.); huangwr@mail.ustc.edu.cn (W.-R.H.); 2 Nanomaterials & Environment Detection Laboratory, Institute of Intelligent Machines, Chinese Academy of Sciences, Hefei 230031, China; mqli@iim.ac.cn

**Keywords:** ultrathin nanowires, nanocomposites, toluene sensing, W_18_O_49_ nanowires

## Abstract

Graphene-based composites have gained great attention in the field of gas sensor fabrication due to their higher surface area with additional functional groups. Decorating one-dimensional (1D) semiconductor nanomaterials on graphene also show potential benefits in gas sensing applications. Here we demonstrate the one-pot and low cost synthesis of W_18_O_49_ NWs/rGO composites with different amount of reduced graphene oxide (rGO) which show excellent gas-sensing properties towards toluene and strong dependence on their chemical composition. As compared to pure W_18_O_49_ NWs, an improved gas sensing response (2.8 times higher) was achieved in case of W_18_O_49_ NWs composite with 0.5 wt. % rGO. Promisingly, this strategy can be extended to prepare other nanowire based composites with excellent gas-sensing performance.

## 1. Introduction

Solid state chemical sensors based on metal oxide semiconductor (MOS) nanomaterials play an important role in the monitoring and detection of gases for environmental applications [1] because MOS’s have the ability to directly interact with the chemical gas traces and subsequently, in determining the gas-sensing properties [2,3,4,5,6]. Moreover, MOS’s have attracted tremendous research interest in gas sensing field due to their properties of superior charge transport, large surface area, and good compatibility and varying conductivity in the presence of a testing gas.

Among the other metal oxides, tungsten oxides, an n-type semiconductor material with a band gap of 2.5–3.0 eV have great significance in many applied fields ranging from gas sensors to solar energy converters, photocatalysts, and electrodes for secondary batteries [7,8,9,10,11,12,13,14]. Lately, tungsten oxide nanocrystals have proven to be promising material for gas-sensing applications because of exceptional sensitivity, excellent stability, and tunable composition [15]. Therefore, various morphologies of tungsten oxides nanocrystals have been widely used as the sensing materials for the detection of various gases such as nitrogen oxides (N_2_O, NO, NO_2_) [16], NH_3_ [17,18], H_2_ [19,20,21,22], ethanol [23], CO [24], H_2_S [25], ozone [26], acetone, and humidity sensing [27].

The sensing ability of gas sensors varies with applied gas concentration and mostly increases with increase in gas concentration. Moreover, the performance of metal oxide gas sensors is influenced by environmental humidity as the water adsorbing on the sensing material surface hampers the electron donation to sensing layers as well as water molecules also act as a barrier against gas adsorption [28,29]. Furthermore, for sensing measurements, various parameters such as stability, sensitivity, selectivity, and response time are firmly related to the MOS gas sensors. So, in order to encounter different detection requirements, many strategies like composition or morphology control [30,31,32], doping composites such as Pd [33], Pt [34], Au [35], and aliovalent doping [36] have been utilized to explore gas sensors with enhanced sensing performance. Among these techniques, carbonaceous materials have appeared as excellent materials to add attractive features into the semiconductor nanostructures due to their unique structure and properties [16]. Graphene has blossomed as a promising candidate for promoting electron transfer due to its large surface area and high intrinsic electron mobility [37]. Graphene based semiconductors composites—such as a-Fe_2_O_3_/rGO [38], ZnO nanosheets/GO [39], Platinum/Graphene Nanosheet/SiC [40], WO_3_ nanorods/G-composites [41], etc.—have been widely reported for the enhanced photocatalytic and gas sensing properties as it not only prevent the agglomeration of nanostructures but also reduces the stacking of graphene sheets that provides more effective surface area for the gas interaction [42]. Moreover, additional synergistic effects between two individual components may be responsible for strong-coupling interaction between graphene and MOS, which are desirable for gas sensing applications. This information would greatly enhance our understanding of multiple roles of graphene in various graphene-semiconductor composites, thereby facilitating the design of multifunction graphene-based composite into gas sensing applications. Other carbonaceous materials such as carbon nanotubes have also been used for gas sensing. Fabian et al. fabricated the gas sensors based on carbon nanotube-ZnO composites for ammonia sensing [43]. Similarly, Rigoni et al. elaborated the very low concentration ammonia sensing at room temperature by using single walled CNTs [44].

Toluene, commonly used chemical reagent is a neurotoxic compound that is almost found at normal working places of artifactitious fields, chemical engineering industry, and in the environment. Therefore, the study of toluene sensing systems is in high demand. Most typical sensors used for toluene gas sensors are surface acoustic wave devices [45], conducting polymers [46], quartz crystal microbalances [47], optical sensors [48], and semiconductor gas sensors [49]. However, among these sensors, the semiconductor based toluene sensors have gained more attention due to their rapid response, good stability, and high sensitivity and many MOS have been used for toluene sensing such as TiO_2_ [50], SnO_2_ [51], WO_3_ [52], and ZnO [53].

In this article, we demonstrate the one-pot synthesis of W_18_O_49_ NWs/rGO composites by simple solvothermal method (the diameter of the nanowires is about 5 nm). Moreover, the gas sensing properties of W_18_O_49_ NWs and W_18_O_49_ NWs/rGO composites on toluene vapors are systematically studied and the effects of rGO on the gas sensing behavior of W_18_O_49_ NWs/rGO composites are discussed briefly. Furthermore, a sensing mechanism for toluene gas detections is also elaborated.

## 2. Materials and Methods

All the chemical reagents used were of analytical grade (Beijing Chemical Co., Ltd., Beijing, China) without further purification.

### 2.1. Synthesis of Graphene Oxide (GO)

GO was prepared from natural graphite powder by a modified Hummers method [54]. Briefly, 5 g of graphite powder was added into the 1 L beaker containing 3.75 g of NaNO_3_ and kept on stirring. A 160 mL measure of sulphuric acid (98%) was gently dropped into the solution and stirred at room temperature. Afterwards, 20 g of KMnO_4_ was poured gradually in a time of 40 min and further kept for stirring for 20 h. After the time period of six days, 500 mL of deionized water (DIW) and 30 mL of H_2_O_2_ (30%) were slowly added into the solution followed by three more days of stirring. The obtained product was passed through the centrifugal washing at 10,000 rpm for 5 min and final product was further purified with dialysis in a week in order to eradicate the remaining salt impurities.

### 2.2. Synthesis of W_18_O_49_ NWs

The synthesis process was carried out by using a one-step solvothermal method with WCl_6_ as precursor and ethanol as solvent [55]. In a typical experiment for the synthesis of W_18_O_49_ NWs, 0.02 g of WCl_6_ precursor was dissolved in 40 mL of ethanol to form a transparent yellow solution. Afterward, the yellow solution was transferred to a PTFE-line 50 mL of autoclave and heated at 180 °C for 24 h. After the reaction completion, the reaction mixture was kept for cooling at room temperature and obtained precipitates were washed with distilled water and centrifuged in ethanol at 4000 rpm for 5 min for purification. The final product was dried under vacuum at 50 °C.

### 2.3. Synthesis of W_18_O_49_ NWs/rGO Composites

The W_18_O_49_ NWs/rGO composites were synthesized by in situ solvothermal method. At first, the 0.02 g of WCl_6_ was dissolved in 20 mL ethanol to obtain a yellow solution. The GO solutions with different amounts of GO were prepared separately in 20 mL ethanol and added to the WCl_6_ solution and stirred for 20 min in order to obtain mixtures in weight ratios of 0.2, 0.5, 1, and 1.5%. Later, the solutions were moved into a PTFE-line 50 mL of autoclave and sustained at 180 °C for 24 h. After the reaction was completed, the reaction mixtures were cooled at room temperature. The obtained precipitates were purified by washing and centrifuged with distilled water and ethanol at 4000 rpm for 5 min. This process was repeated two times and the final product was dried at 50 °C.

### 2.4. Characterization

Transmission electron microscopy (TEM) images were obtained on a JEOL-2010 transmission electron microscope operated at an acceleration voltage of 200 kV. X-ray powder diffraction (XRD) analysis was measured on a Philips X’Pert Pro Super X-ray diffractometer equipped with graphite-monochromatized Cu KR radiation in the 2θ range of 5–80°. The Fourier transform infrared spectroscopy (FT-IR) data was measured on a Thermo Scientific Nicolet iS10 infrared spectrometer. The X-ray photoelectron spectroscopy (XPS) data were measured on ESCALab MKII X-ray photoelectron spectrometer (VG Scientiﬁc, London, UK), using Mg KR radiation as the exciting source. The UV data was measured by using UV-2501PC/2550 (Shimadzu, Tokyo, Japan). The specific surface area was measured with a Quantumchrome ASIQ gas sorption analyzer by degassing the gas under vacuum at 120 °C for 12 h.

### 2.5. Fabrication of Gas Sensors

To prepare the gas sensor device, a certain amount of sample was dispersed in ethanol to form a paste that was deposited onto an alumina ceramic tube with a pair of gold electrodes. To control the temperature, a Ni-Cr wire was inserted into the tube. The paste was dried by heating the tube at 50 °C for 2 h and gas sensing measurements were carried out on a static system. To measure the gas sensing properties, a desired amount of toluene gas was introduced into the system, pre-filled with air and maintained at atmospheric pressure to get various concentrations. The working temperature was controlled by voltage adjustment in order to provide specific current that passed through the Ni-Cr wire, acting as a heater. The electrical measurement was performed and a multimeter was used to monitor the electrical resistance changes.

## 3. Results and Discussions

**Figure 1:**
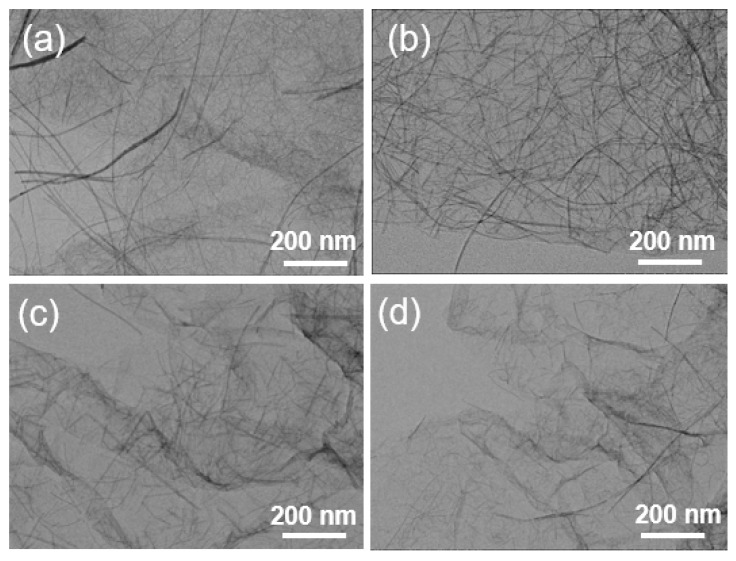
(**a**–**d**) TEM images of W_18_O_49_ NWs/rGO composites with different weight ratios. (**a**) 0.2%, (**b**) 0.5%, (**c**) 1%, (**d**) 1.5%.

**Figure 2:**
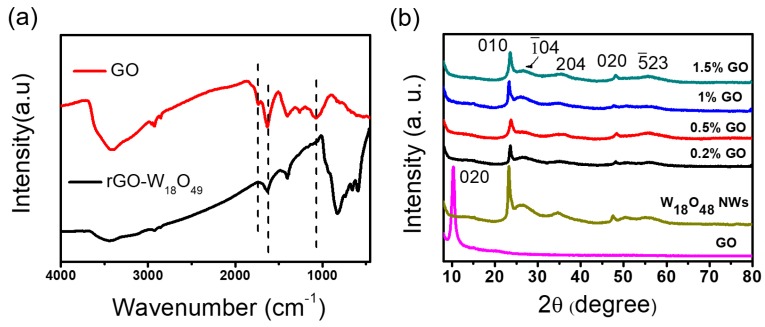
(**a**) FT-IR spectra of GO and 0.5 wt. % W_18_O_49_ NWs/rGO composite (**b**) XRD spectra of the GO, W_18_O_49_ NWs, and W_18_O_49_ NWs/rGO composites with different amount of rGO contents.

Figures S1 and S2 show the transmission electron microscopy (TEM) image of a crumpled layer structure of GO and ultrathin W_18_O_49_ nanowires with a diameter about 5 nm with uniform morphology and large aspect ratio. The TEM images of W_18_O_49_ NWs/rGO composites with different rGO weight ratios (0.2, 0.5, 1, and 1.5 wt. %) are presented in Figure 1a–d, exhibiting the successful assembly of NWs on the surface of graphene sheets due to the presence of functional groups on the GO sheet which provide the nucleation site for the growth of NWs and cause the reduction of GO by W^6+^ ions. Moreover, the addition of GO during synthesis of nanowires does not influence the growth of nanowires as no apparent change in morphology of W_18_O_49_ NWs. The Fourier transform infrared spectroscopy (FT-IR) spectra of GO and 0.5 wt. % W_18_O_49_ NWs/rGO composite are shown the Figure 2a. For the FT-IR spectra of GO, the C=O stretching vibration peak and C-O (alkoxy) stretching peak appear at 1738 cm^−1^ and 1074 cm^−1^ respectively, which disappear in the spectra of 0.5 wt. % W_18_O_49_ NWs/rGO composite, showing the reduction of GO by tungsten salt. The characteristic peaks of W=O and bridging oxygens (OWO) appear in the region of 1000–500 cm^−1^ [56] which can be seen in the IR spectra of 0.5 wt. % W_18_O_49_ NWs/rGO composite. The peak at 1628 cm^−1^ is allocated to the skeletal vibration of graphene sheets [41].

The X-ray diffraction (XRD) spectra shown in Figure 2b confirms the successful formation of W_18_O_49_ NWs/rGO composites. The XRD spectra of GO is presented in Figure S3, showing a strong peak at the 2θ position of 11.22° which corresponds to the (002) interlayer [57]. Figure 2b shows the XRD spectra of W_18_O_49_ NWs/rGO composites with different rGO weight ratios of 0.2, 0.5, 1, and 1.5 wt. %. The XRD patterns of W_18_O_49_ NWs/rGO composites show similar spectra to that of pure W_18_O_49_ NWs (Figure S4) and can be indexed to monoclinic structure type (P2/m) W_18_O_49_ (JCPDS: 84-1516) with cell constant of a = 18.318, b = 3.782, and c = 14.028 Å. The characteristic peaks appearing at 2θ values of 23.65°, 26.31°, 34.91°, 47.55°, and 55.74° match the (010), ($\bar{1}04$), (204), (020), and ($\bar{5}23$) planes of W_18_O_49_ NWs respectively and exhibit the preferential growth of the W_18_O_49_ crystals along the (010) direction due to high intensity of (010) plane, showing successful formation of W_18_O_49_ NWs which is similar behavior to the previously reported literature [55]. No characteristic peak related to rGO is observed due to the low concentration and low diffraction intensity during the comparison with the W_18_O_49_ NWs peak that appears between 20° and 30° [41,58].

**Figure 3:**
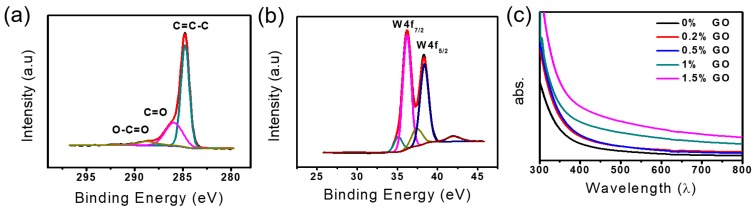
(**a**) XPS spectra of 0.5 wt. % W_18_O_49_ NWs/rGO composite, (**b**) W4f core-level spectra of 0.5 wt. % W_18_O_49_ NWs/rGO composite, (**c**) UV–vis spectra of W_18_O_49_ NW and W_18_O_49_ NWs/rGO composites (0.2, 0.5, 1, and 1.5 wt. %).

For the identification of changes in the functional groups, core level C 1s spectra are examined using X-ray photoelectron spectroscopy (XPS) analysis. The XPS spectrum of GO is presented in Figure S5, showing four different peaks at the position of 284.7, 286.9, 287.8, and 288.6 that belongs to the C–C/C=C, C-O, C=O, and O–C=O bonds, respectively [59,60]. After the accumulation of W_18_O_49_ NWs on rGO (Figure 3a), the C 1s XPS spectrum of composite shows an increase in intensity of C–C/C=C peak from 46.1% to 74.3% whereas the peak intensities of oxygen-containing functional groups are decreased, indicating the significant restoration of sp^3^/sp^2^-hybridized carbon structures [38]. The hydroxyl peak is observed in the spectrum of 0.5 wt. % W_18_O_49_ NWs/rGO composite at the position of 285.9, showing the same phenomena as reported before [61,62]. This generation of OH peak is due to the ring opening reaction of epoxides, commonly known as the reduction products of the oxygen-containing functional groups [38]. The high resolution spectra of W4f for 0.5 wt. % W_18_O_49_ NWs/rGO composite shows two peaks 35.7 and 38.1 eV that can be attributed to W4f_7/2_ and W4f_5/2_, respectively (Figure 3b) [55]. Moreover, the binding energies of W4f_7/2_ and W4f_5/2_ for the 0.5 wt. % W_18_O_49_ NWs/rGO composite are moved to lower values, indicating the interaction between W_18_O_49_ NWs and the rGO during the formation process of the W_18_O_49_ NWs/rGO composites [63].

**Figure 4:**
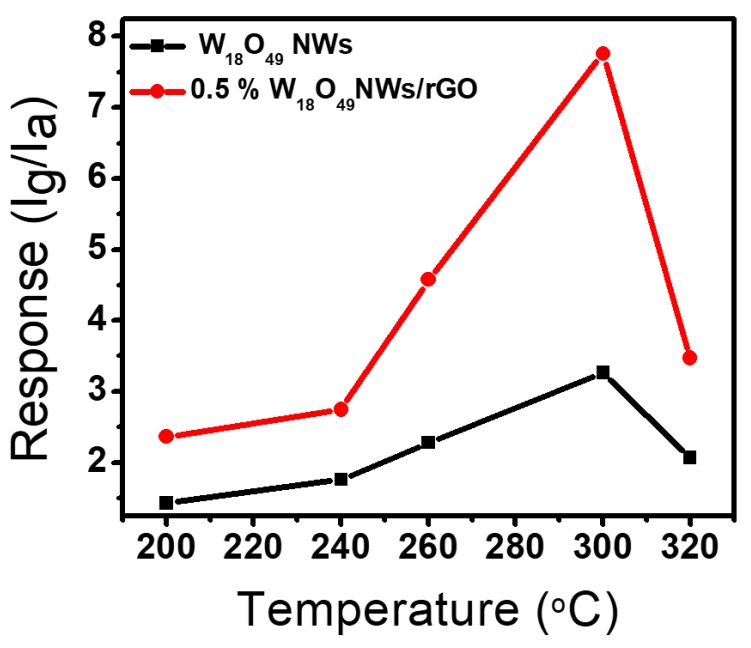
Gas sensing response of pure W_18_O_49_ NWs and 0.5 wt. % W_18_O_49_ NWs/rGO composite towards 100 ppm toluene vapors at different temperature.

The UV–visible absorption spectra of pure W_18_O_49_ NWs and W_18_O_49_ NWs/rGO composites with different rGO contents (0.2, 0.5, 1, and 1.5 wt. %) are presented in Figure 3c. It was observed that by introducing different amount of graphene not only enhance the intensity of absorption but also shifted the light absorption range towards visible area [64]. The Brunauer–Emmett–Teller (BET) measurements for the determination of surface area are presented in Figure S6a–c as surface area is a key factor for good efficiency of sensing materials. For the enhanced surface area measurement by the addition of GO, the specific surface area of pure W_18_O_49_ NWs and 0.5 wt. % W_18_O_49_ NWs/rGO composite were measured through the nitrogen adsorption–desorption tests. The BET surface area of pure W_18_O_49_ NWs and 0.5 wt. % W_18_O_49_ NWs/rGO composite was 72.843 m^2^/g and 81.843 m^2^/g, indicating the increase in surface area of 0.5 wt. % W_18_O_49_ NWs/rGO composite due to rGO addition. The gas detection applications were demonstrated by fabricating a number of gas sensor devices based on pure W_18_O_49_ NWs and W_18_O_49_ NWs/rGO composite comprising various amount of rGO ranging from 0.2 wt. % to 1.5 wt. %. The sensing behavior of most of metal oxide gas sensors is highly dependent on operating temperature. So at first, the temperature dependent gas sensing properties were investigated by operating gas sensors at different temperature and at a specific gas concentration. Figure 4 presents the gas sensing response of pure W_18_O_49_ NWs and 0.5 wt. % W_18_O_49_ NWs/rGO composite to 100 ppm toluene vapors as a function of operating temperature. The response of a sensor was defined in terms of current ratio (S = I_g_/I_a_, where I_g_ = sensor current in gas environment and I_a_ = sensor current in air). However, the sensing response factors such as response time and recovery time were calculated by time taken for the sensor to attain 90% of the total current change in the case of adsorption and desorption of gas, respectively. The increasing temperature resulted the increase in sensing response for 100 ppm toluene vapors and maximum sensing response for both pure W_18_O_49_ NWs and 0.5 wt. % W_18_O_49_ NWs/rGO composite to 100 ppm toluene vapors was observed at 300 °C. Afterwards, a gradual decline in response was obtained by increasing the temperature at 320 °C. Hence optimum working temperature for gas sensing measurements was selected at 300 °C. The increase in sensing response at higher temperature is due to enough thermal energy to react with the surface adsorbed oxygen species. Whereas, very high temperature results in a decrease in sensing response due to the difficulty in gas adsorption and low utilization rate of the sensing layer [65,66].

**Figure 5:**
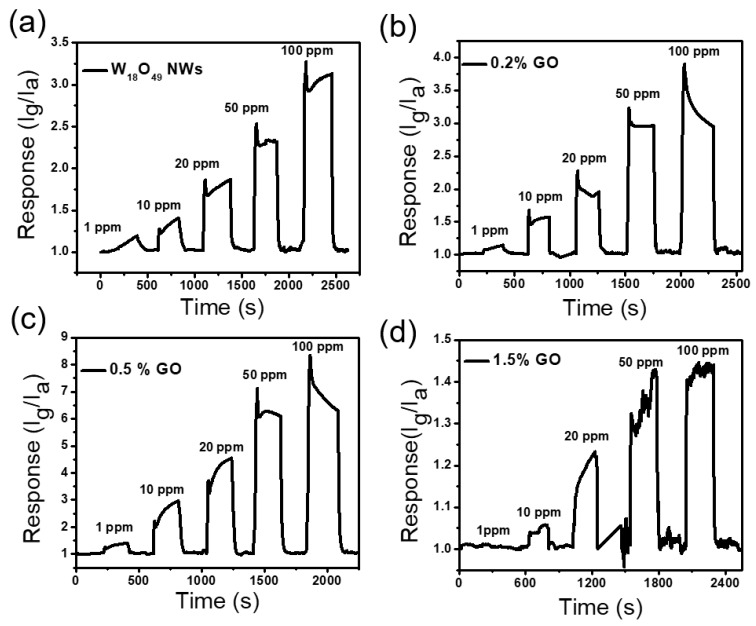
Gas sensing properties of various samples at 300 °C towards toluene vapors. (**a**) pure W_18_O_49_ NWs, (**b**) 0.2 wt. % W_18_O_49_ NWs/rGO composite, (**c**) 0.5 wt. % W_18_O_49_ NWs/rGO composite, (**d**) 1.5 wt. % W_18_O_49_ NWs/rGO composite.

The response of sensors towards various concentrations of toluene vapors was investigated at 300 °C in order to study the dynamic range of sensors. Figure 5a–d presents the sensing response of sensors based on pure W_18_O_49_ NWs and 0.2, 0.5, and 1.5 wt. % W_18_O_49_ NWs/rGO composites respectively, from 1 to 100 ppm concentration of toluene vapors. The sensor response increases to a steady value with increase in gas concentration and fall to initial value upon injecting air, revealing good response and recovery features. As compared to pure W_18_O_49_ NWs, the W_18_O_49_ NWs/rGO composites with 0.2 and 0.5 wt. % rGO show a better response to different concentrations of toluene vapors and high response is achieved in the case of 0.5 wt. % W_18_O_49_ NWs/rGO composite (Figure 5c). However, the sensitivity of sensors is decreased by using an amount of GO higher than 0.5 wt. % and sample containing 1.5 wt. % of rGO shows poor efficiency to toluene sensing (Figure 5d). This low sensing efficiency can be attributed to the high conductivity of composites that can certainly reduce the resistance variation of composite.

**Figure 6:**
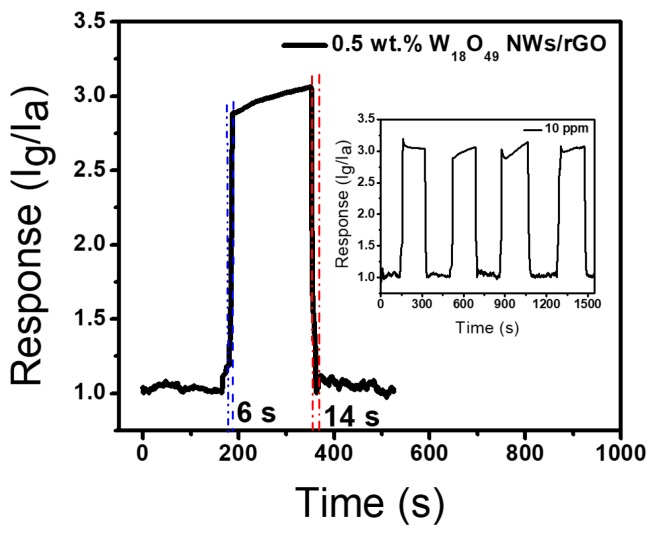
Response and recovery time measurement of sensor based on the 0.5 wt. % W_18_O_49_ NWs/rGO composite to 10 ppm toluene vapor at 300 °C. The inset is the repeatable response–recovery curves to 10 ppm toluene vapor at 300 °C.

Figure 6 shows the response and recovery features of the sensor based on the 0.5 wt. % W_18_O_49_ NWs/rGO composite to 10 ppm toluene vapor at 300 °C. Upon exposure to toluene vapors, the current increases and reaches to steady state value merely in 6 s. Afterward, by injecting air the current of sensor decreases and recovers to its initial value in 16 s. Figure 6 (inset) displays the repeated response–recovery curves of the sensor, showing good sustainability of sensors to its initial response amplitude upon four cyclic tests and reveals the good repeatability of the sensor. Moreover, the response and recovery time of W_18_O_49_ NWs and 0.5 wt. % W_18_O_49_ NWs/rGO composite towards toluene vapors with varying concentrations are presented in Figure S7, showing quick response in case of 0.5 wt. % W_18_O_49_ NWs/rGO composite.

**Figure 7:**
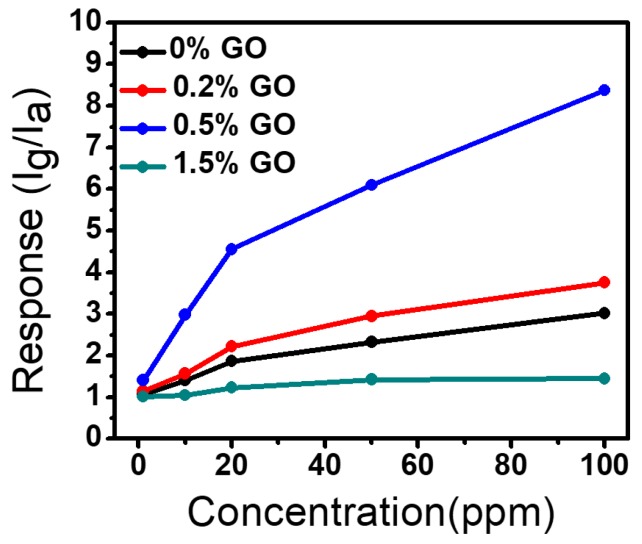
Linear gas response of W_18_O_49_ NWs and W_18_O_49_ NWs/rGO composites towards different concentrations of toluene vapors at 300 °C.

Figure 7 presents the linear relationship of sensing response of samples towards various toluene gas concentrations. The response of all samples increases almost linearly with increases in gas concentration and the linear response is observed above 20 ppm concentration. The detection limit of sensors is around 1 ppm with a sensing response of 1.06, 1.146, 1.404, and 1.011 for W_18_O_49_ NWs and W_18_O_49_ NWs/rGO composites (0.2, 0.5, and 1.5 wt. %), showing high response value of 0.5 wt. % W_18_O_49_ NWs/rGO composite. According to IUPAC definitions the signal can be true if the signal to noise ratio is higher than 3 [67], so the signal to noise ratio of 1 ppm data was measured which obtained around 7.305, showing the stability of signal at very low concentration. The response of pure W_18_O_49_ NWs, and W_18_O_49_ NWs/rGO composites (0.2, 0.5, and 1.5 wt. %) at 100 ppm toluene vapors are 3.02, 3.75, 8.375, and 1.447, respectively, showing a 2.8-fold increase in sensing response in case of 0.5 wt. % W_18_O_49_ NWs/rGO composite as compared to the pure W_18_O_49_ NWs.

The selectivity of sensors was investigated by injecting different gases (methanol, ethanol, toluene, and formaldehyde) to 0.5 wt. % W_18_O_49_ NWs/rGO composite sensor, showing the strongest response towards toluene among other gases (Figure S8). While comparing the sensing characteristics with pure W_18_O_49_ NWs, the graphene based composites show enhanced sensing properties and exhibit a 2.8-times increase in sensing response at 100 ppm toluene concentration. The presence of graphene not only facilitates the improved conductivity of composites but also increases the surface area for gas adsorption as discussed above (Figure S5a–c). Moreover, a P–N junction is formed due to p and n type nature of graphene and W_18_O_49_ NWs respectively, which improves the transfer of electrons from NWs to graphene sheet. Upon exposure to air, more oxide formations (O^2−^, O_2_^−^, and O^−^) occur by the adsorption of atmospheric oxygen due to the additional surface area and high electron density. The oxide formation increases the Schottky barrier and also the electrical resistance. On the contrary, in toluene atmosphere, the reaction between toluene molecules and oxygen species will release electrons and decrease the electrical resistance. Hence, the enhanced surface area and improved electrical properties due to the presence of graphene upgrade the sensing properties of W_18_O_49_ NWs/rGO composites for their effective utilization in gas sensing.

## 4. Conclusions

In summary, a one-pot solvothermal method was developed for the controlled synthesis of rGO-W_18_O_49_ NW composites. A relative gas-sensing study was performed by fabricating a number of sensors based on pure W_18_O_49_ NWs and W_18_O_49_ NWs/rGO composites containing rGO in different weight ratios of 0.2, 0.5, and 1.5 wt. %. The W_18_O_49_ NWs/rGO composites containing suitable amount of rGO revealed improved gas sensing response to toluene vapors and the composite containing 0.5 wt. % of rGO showed a highest response at 300 °C and 100 ppm toluene vapors that was almost 2.8-fold higher than pure W_18_O_49_ NWs. This study provides a potential approach to enhance the gas sensing response of W_18_O_49_ NWs.

## Supplementary Materials

The following are available online at http://www.mdpi.com/1424-8220/17/10/2245/s1, Figure S1: TEM image of GO. Figure S2: TEM image of W_18_O_49_ NWs. Figure S3: XRD spectra of GO. Figure S4: XRD spectra of W_18_O_49_ NWs. Figure S5: XPS spectra of GO. Figure S6: W_18_O_49_ NWs and 0.5wt% W_18_O_49_ NWs/rGO composite. Figure S7: Response and recovery time of the sensors. Figure S8: Gas sensing response with different gases.
