# Supplementary material for: Ultrathin Tungsten Oxide Nanowires/Reduced Graphene Oxide Composites for Toluene Sensing
Source: Sensors (Basel). 2017 Sep 29;17(10):2245. doi: 10.3390/s17102245 (PMC5677425; doi:10.3390/s17102245)
Supplement: Supplementary file 1 [file sensors-17-02245-s001.pdf]

Ultrathin Tungsten Oxide Nanowires/Reduced Graphene Oxide Composites for Toluene Sensing

Muhammad Hassan[1], Zhi-Hua Wang, Wei-Ran Huang, Min-Qiang Li[2], Jian-Wei Liu, Jia-Fu Chen*

[1] Hefei National Laboratory for Physical Sciences at the Microscale, Collaborative Innovation Center of Suzhou Nano Science and Technology, Department of Chemistry, University of Science and Technology of China, Hefei 230026, China

[2] Chinese Acad Sci, Inst Intelligent Machines, Nanomat & Environm Detect Lab, Hefei 230031, China

* Correspondence: jfchen@ustc.edu.cn; Tel.: +86-551-6360-1195

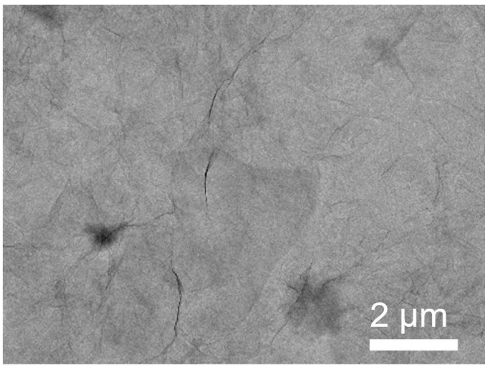

Figure S1 TEM image of GO.

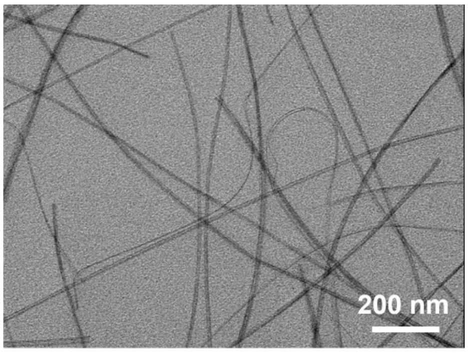

Figure S2 TEM image of $W_{18}O_{49}$ NWs.

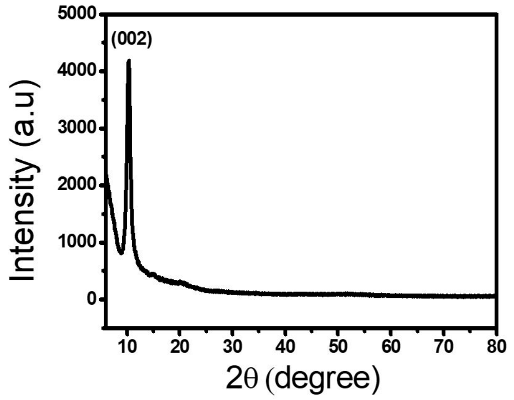

Figure S3 XRD spectra of GO.

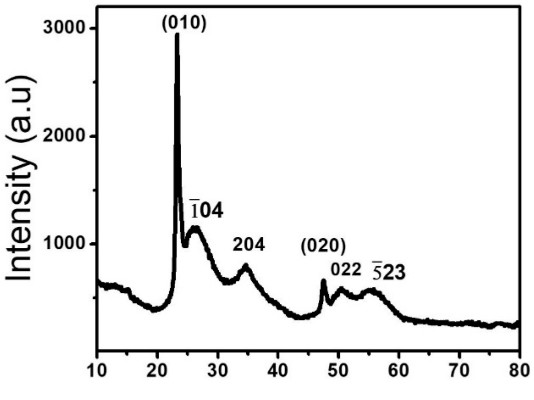

Figure S4 XRD spectra of $W_{18}O_{49}$ NWs.

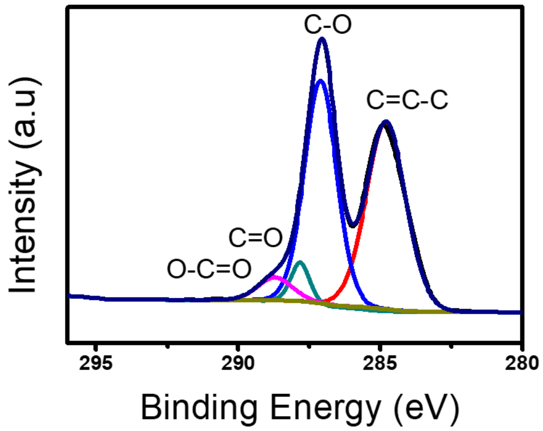

Figure S5 XPS spectra of GO.

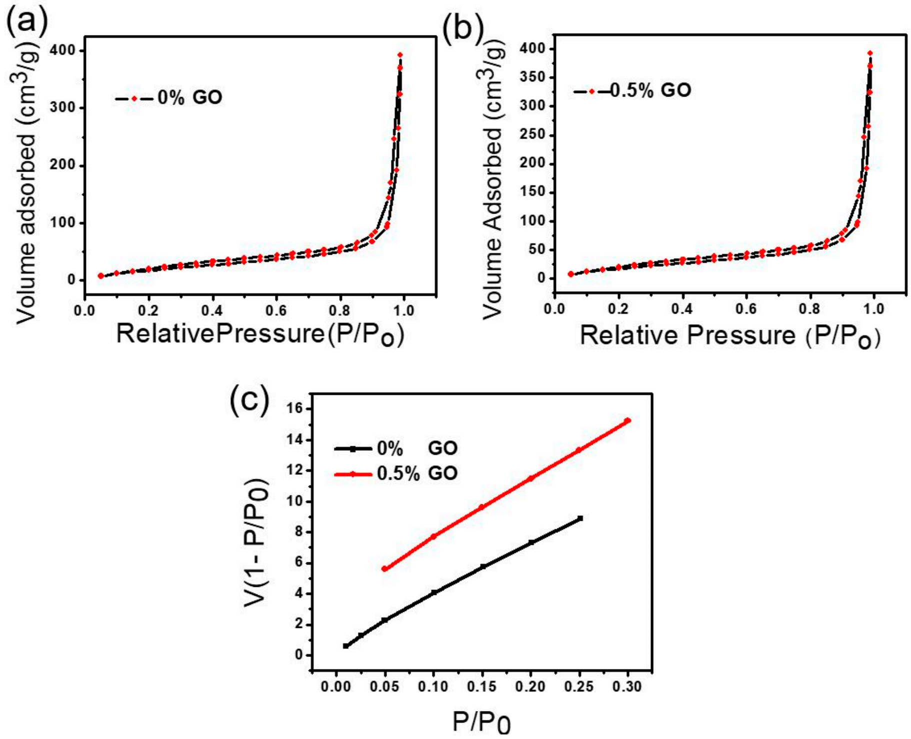

Figure S6 (a, b)Nitrogen adsorption-desorption isotherm curves of $W_{18}O_{49}$ NWs and 0.5wt% $W_{18}O_{49}$ NWs/rGO composite, (c) linear form BET isotherm of Pure $W_{18}O_{49}$ NW and 0.5wt% $W_{18}O_{49}$ NWs/rGO composite.

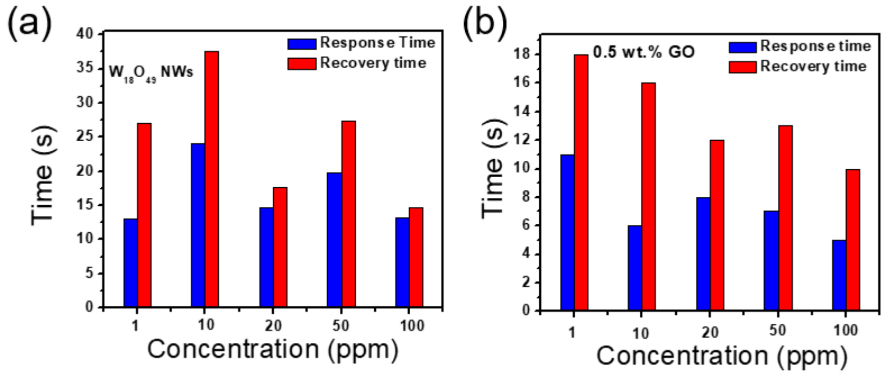

Figure S7. Response and recovery time sensor based on (a) $W_{18}O_{49}$ NWs and (b) 0.5 wt% $W_{18}O_{49}$ NWs/rGO composite to different concentrations of toluene vapor at 300 ˚C.

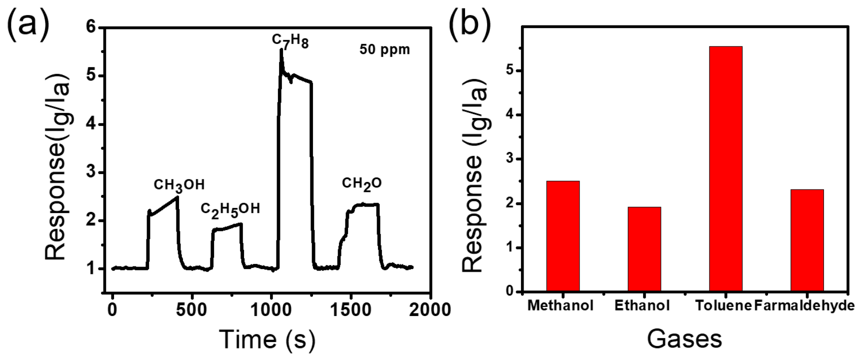

Figure S8 Gas sensing response of 0.5 wt.% $W_{18}O_{49}$ NWs/rGO composite towards 50 ppm methanol, ethanol, toluene and farmaldehyde gases.
